# Design, development and evaluation of a mobile-based self-care application for patients with COVID-19 not requiring hospitalization; a study of Northwest of Iran

**DOI:** 10.1186/s12911-023-02381-3

**Published:** 2023-12-06

**Authors:** Mohammad Heydari, Esmaeil Mehraeen, Elham Javaherikiyan, Nahid Mehrabi, Mostafa Langarizadeh, Vahideh Aghamohammadi, Hamed Rezakhani Moghaddam, Khadijeh Nasiri

**Affiliations:** 1 Department of Health Information Technology, Khalkhal University of Medical Sciences, Khalkhal, Iran; 2 Health Information Technology, Social Security Organization, Tehran, Iran; 3 Department of Health Information Technology, Aja University of Medical Sciences, Tehran, Iran; 4 Department of health information management, School of health management and information sciences, Iran university of medical sciences, Tehran, Iran; 5 Department of Nutrition, Khalkhal University of Medical Sciences, Khalkhal, Iran; 6 Department of Public Health, Khalkhal University of Medical Sciences, Khalkhal, Iran; 7 Department of Nursing, Khalkhal University of Medical Sciences, Khalkhal, Iran

**Keywords:** Mobile-health, Self-care, COVID-19, Mobile app, Evaluation

## Abstract

**Background:**

Given the effective role of a mobile applications in disease management, disease monitoring, and self-care in patients with COVID-19 disease, we aimed to design, development and evaluation of a self-care Mobile app for COVID-19 patients not requiring hospitalization.

**Methods:**

The design, development and evaluation the usability of the self-care and education mobile app for patients with COVID-19 disease were conducted in two main phases at 2021 in Northwest of IRAN; (1) Determine the features and capabilities and (2) Design, development and evaluation of self-care mobile App. JAVA programming languages and Android Operating System were used and selected to design and development of a mobile app. There were 25 participants who conducted evaluations of the mobile app’s usability and impact using the mobile health app usability a Questionnaire of User Interface Satisfaction was administered to assess the usability of the developed application. The results were analyzed via Excel 2013.

**Results:**

The model of developing a mobile app as an Information System was the Waterfall model. The smartphone application based on a set of capabilities and features was designed and consists of two main parts: the login screen for user registration, and the main home menu. The user interface includes three main pages or activities; (a) Main Menu for quick access to all of the pages, (b) Symptom management and monitoring to monitor the signs and symptoms during the illness, and (c) Set Reminders and Alarms to notify patients. The users’ mean score of the application usability was calculated as 7.91 out of 9 indicating a good level of satisfaction.

**Conclusion:**

This app can be a guideline and a useful tools for managing and monitoring symptoms, reminding medications, and implementing self-care instructions in outpatients. The authors suggest evaluating the efficacy and functionality test of mobile-based applications for COVID-19 in clinical trial studies.

## Background

SARS-CoV-2 led to the outbreak of severe respiratory disease (COVID-19) in Wuhan, China, which is spreading rapidly around the world [1–3]. Adherence to strict hygiene rules and proper treatment to protect against respiratory viruses is a vital issue [4]. Among the ways to reduce disease transmission are quarantine and reducing social interactions [5, 6]. Therefore, given the quarantine and public need, the level of public awareness about COVID-19 should be increased [7]. Indeed, people’s awareness and skills in self-care should be strengthened [8].

The COVID-19 pandemic situation and the spread of the virus depend on the awareness and behavior of the self-care instructions of people. For example, people should be familiar with social interaction guidelines, home isolation, adherence to preventive measures symptom recognition, and care [9, 10]. In an epidemic, knowing the severe symptoms, how to measure them, and how to act according to the latest guidelines can be a challenge for many people. Lack of awareness and compliance with self-care instructions in Iran was one of the major challenges and limitations in COVID-19 pandemic management [11–13]. Self-care has five sequential processes including positive health measures, the process of early diagnosis, monitoring of symptoms, follow-up treatment, and evaluation of treatment effectiveness [14, 15]. Unique and innovative solutions are needed to meet the basic needs of patients with COVID-19 and others in need of health care [16]. In this sense, technological advances provide new options [17, 18]. One of the most effective ways to improve health and provide essential services to people in order to control COVID-19 is through digital technologies, especially mobile applications [19, 20].

Mobile applications (herein as mobile apps) are designed and developed to run on smartphones with special usage [21, 22]. Today, smartphones and mobiles have been accessed easily by most people, and smartphones are a candidate platform for delivering and retrieving health information [23]. Today, many patients, physicians, nurses, and healthcare staff are interested in mobile apps [24, 25]. Across healthcare, the potential of mobile apps is enormous [26]. Mobile Health Apps have a variety of applications in the COVID-19 epidemic, including real-time tracking of the disease [27, 28] and its symptoms to identify connections between people suspected of having the virus [29]. Other uses of mobile health apps include providing remote health services, including diagnosis [30], counseling [31], as well as treatment and management services [32]. Adherence and monitoring to Self-care instructions by assisting smartphones apps are part of the useful usages of mobile health apps [33]. Therefore, due to the need to reduce social interactions during the COVID-19 epidemic [34, 35], mobile health apps can be effective and efficient in preventing disease spreading, adherence to self-care instructions, and even self-management [36, 37].

Mobile health apps can be a practical and attractive way to improve self-management [38]. In particular, mobile health app interventions are provided to facilitate patient-care provider communication, health literacy, health information exchange, support decision-making, and peer support, without the time and geography constraints, all of which are important for self-care [39–41]. Using this technology in the epidemic era can lead to improving disease control and clinical case management [42, 43] and People who are in quarantine can use these tools to take care of themselves [44]. Mobile health apps usually effectively improve the quality of care and can be adapted quickly on a large scale and at a low cost [45]. The advantages of mobile health app interventions include ease of use, scalability, personalization, and the ability to “send time-sensitive messages” with an always-on device [46–48]. Therefore, this technology is an attractive, effective, and cost-effective option for managing the pandemic [49]. As aforementioned, the dispersion of the necessary education related to COVID-19 and the lack of an integrated environment of self-care instructions was one of the main problems of the COVID-19 pandemic in Iran. Therefore, considering the benefits of using mobile health apps in the self-care of patients with COVID-19, in this study, the design, development and usability evaluation of a mobile-based self-care application for patients with COVID-19 not requiring hospitalization has been conducted.

## Methods

This study was an applied development study and is partial to a large study plan that was carried out and conducted in four main phases. The present study is related to the second phase which was an applied and developmental study to design, develop, implement and evaluation of a mobile-based education and self-care application for patients with COVID-19 disease not requiring hospitalization conducted in 2021 and 2022 in Northwest of Iran. The study method was based on the developing of a mobile health app including UI design and backend programming and also usability evaluation. This app is used to support patients not requiring hospitalization during their treatment at home by educating them, providing self-care instructions, symptom monitoring, and tracking. This educational and self-care mobile health app for COVID-19 is fully stand-alone and is not connected to healthcare providers or electronic health records. The design, development and evaluation of the COVID-19 self-care mobile app were done in two main phases:

### Determine the features and capabilities

To identify and determine the features and capabilities of the self-care mobile health app for patients with COVID-19 disease not requiring hospitalization, a checklist of the required capabilities of the self-care instructions was prepared and provided to the experts and physicians of the clinical and technical team in order to determine these features [21, 50]. After identifying key features, we designed a conceptual model to create a prototype of a mobile app. Based on key features of similar mobile health apps that were found, this model was applied in three parts, first user registration, second educating common information about COVID-19 and self-care instructions to patients, and third symptom monitoring and tracking daily in this mobile health app.

### Design, development and usability

The Waterfall Model was used to design and develop the COVID-19 self-care mobile health app. This stage consisted of drawing a conceptual model of the user’s relationship with the app; designing the prototype, releasing the initial version, and finally publishing the original version. Based on the results of the first phase to develop a self-care mobile health app, a conceptual and flowchart model of the overall trend was designed. According to this model and using the Java programming language in the integrated development environment (IDE) of Android Studio V 2.3.3, a prototype of a mobile health application was created. In the operational section, Java object-oriented programming language to develop the back-end of the app, classes, and methods; to create the database SQLite DB; and to design the layouts and user interface of the app, expandable Markup Language (XML) was employed. The mobile app sends information after receiving it from the user to the Central database. After entering user information, the user saves them and can also edit profile information. Android Volley library was used for networking requests to handle. Volley is a library that makes networking for Android apps easier and most importantly, faster. Due to the popularity of the Android operating system in Iran, the initial version of the self-care mobile health apps was designed for the Android operating system version 4.4 KitKat and above [25, 51].

After designing the application, its usability was assessed by users. To complete this stage 30 patients with Covid-19 were invited to evaluate the mobile app from which 25 were accepted to participate in the evaluation stage. First, the application was installed on the participants’ smartphone devices. Secondly, the participants were asked to use the application over two weeks. Finally, participants were asked to express their opinions about the mobile apps via Questionnaire for User Interaction Satisfaction (QUIS) (version 5.5). The validity and reliability of this questionnaire have been confirmed [52, 53]. This questionnaire was designed based on the 10-point Likert scale. The questionnaire consists of 6 sections; section one is for the Overall reaction to the app; Section two is about screen software; Section three is about Terminology and information used in the application; Section four is about Learning; Section Five for App capabilities and section six is total opinin of users about the mobile app or software. In this questionnaire, scores are classified between 0 and 9. Mean scores of 0–3 were classified as poor; 3–6 as intermediate, and 6–9 as good. The results at this stage were analyzed by means of descriptive statistics in Excel 2013 software, where means and standard deviation were also calculated.

### Informed consent and ethical considerations

This study got an Ethics Number by the Medical Ethics Committee of the Khalkhal University of medical sciences with the reference number IR.KHALUMS.REC.1399.010. Informed consent form was obtained from all participants or legal guardian after explaining the objectives of the study. We confirm that all methods were performed in accordance with the relevant guidelines and regulations.

## Result

### User interface design process

**Fig. 1:**
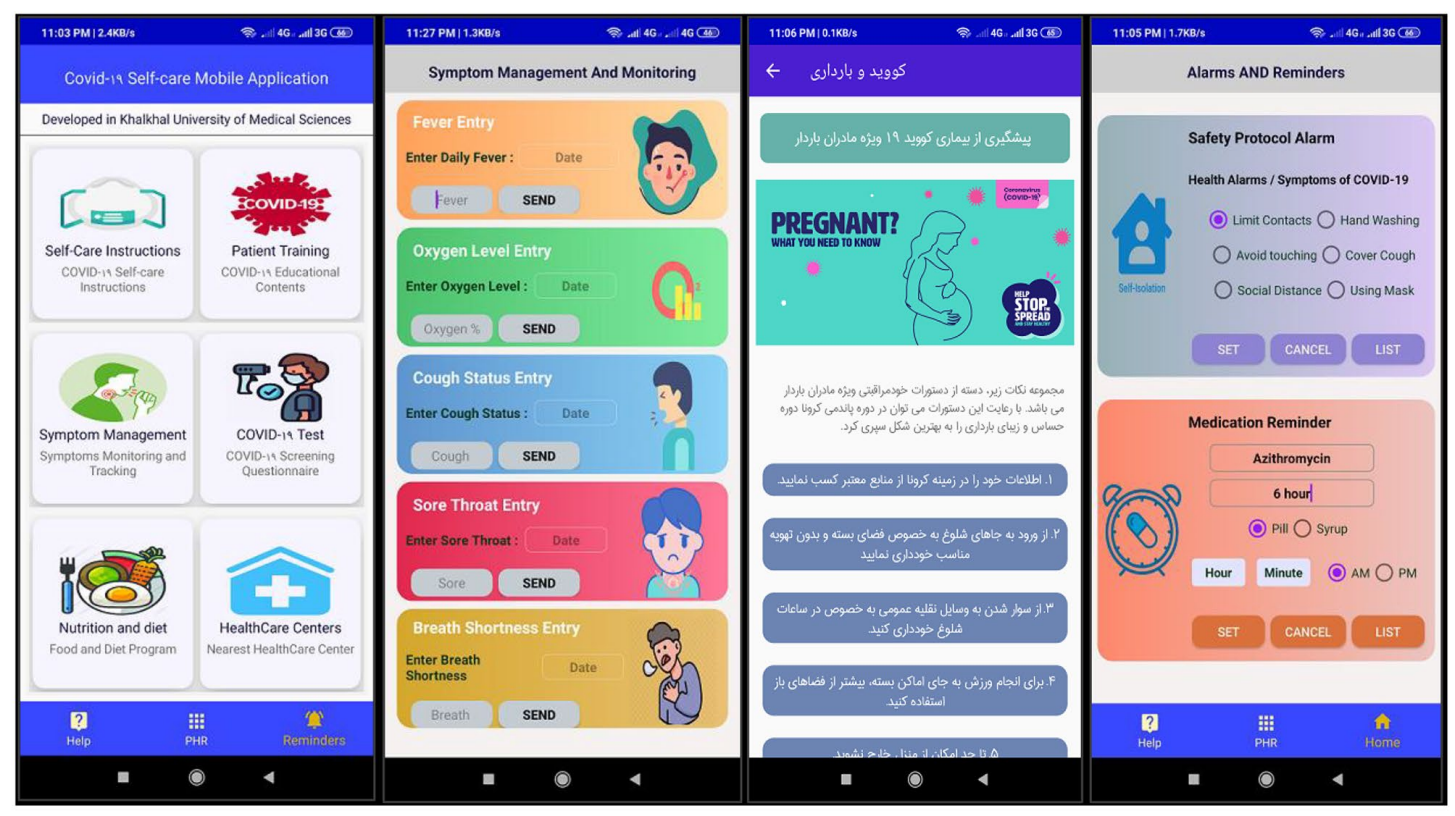
Screenshots of the Mobile app (i.e. The User Interface of COVID-19 Self-care Mobile Application) From top Left to Right bottom; (**a**) Main menu page, (**b**) Set Symptom Management & Monitor, (**c**) Self care instruction for pregnant women (**d**) Set Reminders and Alarm page

We used extensible markup language (XML) to design the user interface and layouts. Given that the easiest and most productive mechanism for designing a user interface for a mobile app is Android Studio software, we used the Integrated Development Environment (IDE) of Android Studio as a designer tool to design UI. For creating an elegant UI newest Material Designs in Android Studio were used. Material Design is a visual language that synthesizes the classic principles of good design with the innovation of technology and science. The user interface was include three main pages or activities; (a) Main Menu or home page, (b) Symptom management and monitoring, (c) Self care instructions for pregnant women, and (d) Set Reminder and Alarms. A set of mobile app pages or activities are shown in Fig. 1.

### The architecture

**Fig. 2:**
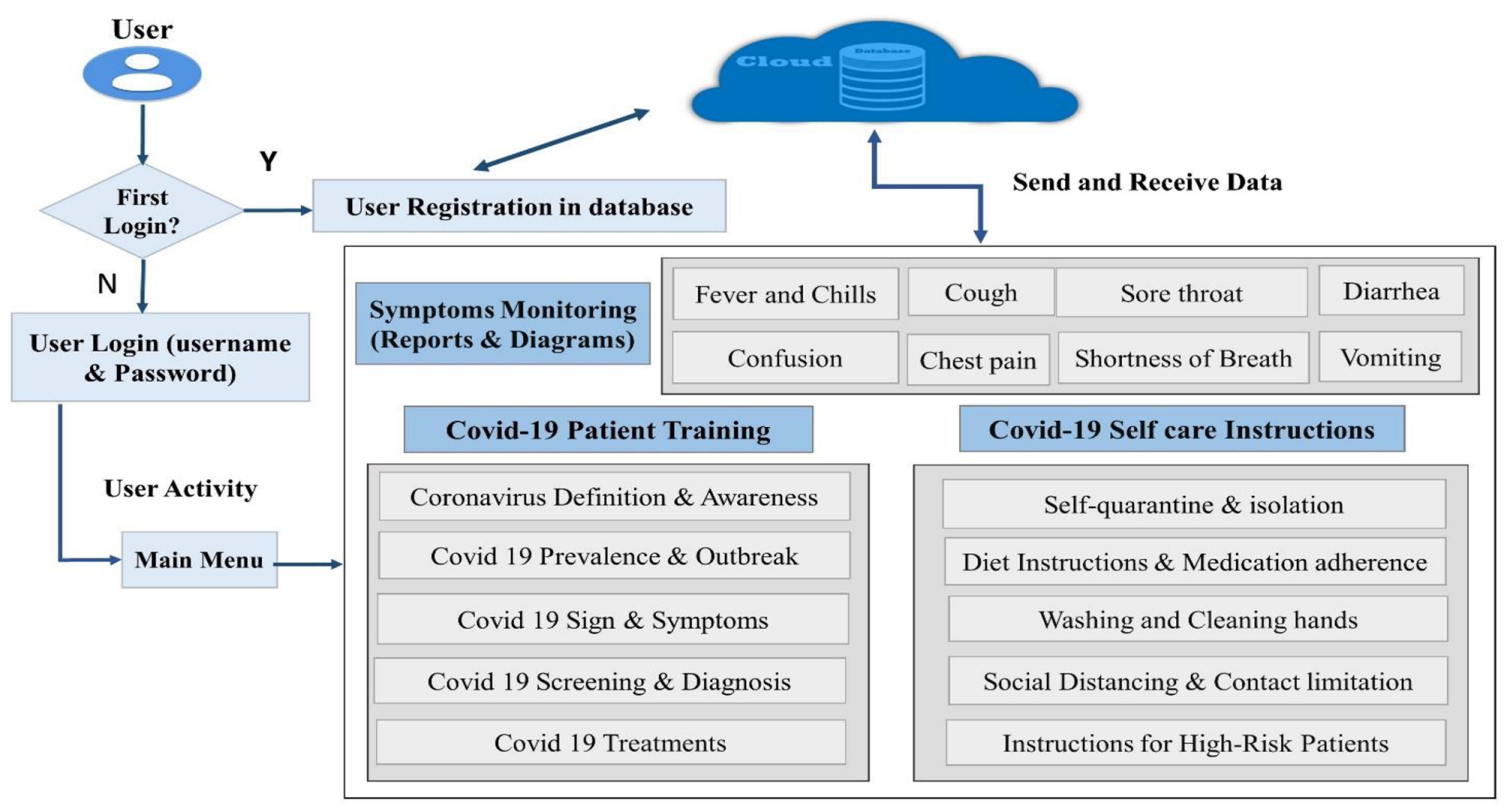
The Architecture of the COVID-19 Self-care Mobile App

The Waterfall Model was used to design the mobile app. In the first step planning and requirements were determined. In the next step, designing was done, and in the third step, the mobile app was installed and implemented on users’ smartphones; In the last step, the usability evaluation was conducted. The main architecture of COVID-19 self-care at the two steps for designing the mobile app is provided based on a previous study and is shown (Fig. 2). In general, after Logging in, patients may either search for new educational concepts about Covid 19, in the monitoring section enter the new status of symptoms and view daily reports, and also set reminders for drugs or appointments with a doctor. User for the first login must create an account, definition username, and password, after launching and installing the app; The user after entering the app can register the profile information and clinical status in a personal health record (PHR); the clinical status includes the information related to his underlying illnesses and health. The registration screen (as well as a login screen) is designed to authenticate the users by sending a verification SMS.

In the main menu of the COVID-19 self-care mobile app, based on the design methods and user interfaces, all the sections required by the user for easy access were placed in layouts. Object-Oriented Programming (OOB) with JAVA programming languages ​​was used to develop the backend of the Covid-19 self-care mobile app. With the flourishing presence of the Android Operating System (AOS) and the widespread use of Android-based mobile smartphones in Iran; this Operating System was selected and chosen as a platform for the COVID-19 self-care mobile health app. To receive and send data, the Volley library has been used to automate the network operations. The architecture of the Covid-19 self-care mobile app is shown in Fig. 2.

### The model and key features

**Fig. 3:**
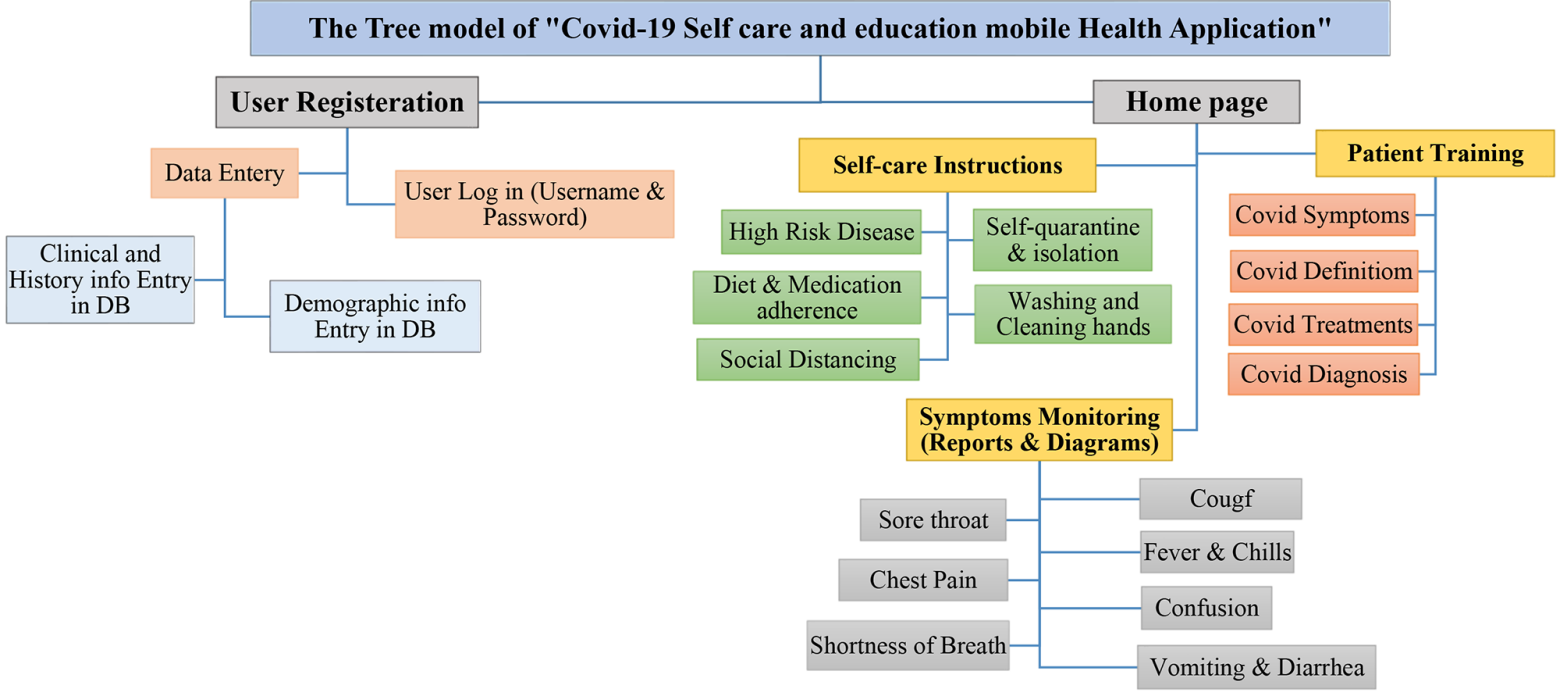
The Tree Model of the COVID-19 Selfcare Mobile health App for Patients

The main model and a set of features and capabilities of a self-care mobile app related to COVID-19 are presented in Fig. 3. Based on the main model provided, the app consists of two main parts: the user registration and login page and the main home page. At the first use of the app, simultaneously with installing and registering in the app, users in each activity and section get helped and guided by dialogue message boxes. After these introductory steps, the user would enter the home page and main menus and can choose options including (1) COVID-19 educational topics, (2) COVID-19 self-care instructions; (3) COVID-19 symptom management, and (4) set Reminders and alarms.

When epidemics like coronavirus occur, it is important to be aware and knowledgeable about the disease and virus behaviors. Knowing signs and symptoms, diagnosis and treatment ways, and self-care instructions seem necessary and useful. In the educational part of the application, training about coronavirus, methods of diagnosis, treatment, and control of the disease are mentioned. In the COVID-19 symptom management section, patients are allowed to manage their symptoms such as cough, fever and chills, chest pain, shortness of breath, sore throat, confusion, etc. Monitoring and tracking symptoms with reports and diagrams will be shown to patients.

Another feature of this COVID-19 self-care mobile app is reminders and alarms. Patients can set reminders for drugs and other activities, and adherence to taking medication can be registered in the app. Alerts in the app if there are rising fever, cough, or other signs that become abnormal are another feature of the app and alerts will be displayed on alert dialogue with information to be seen. The architecture of the mobile app and the flow of information between the mobile app and the cloud-based server in Fig. 2 are illustrated. All the patient’s data is deployed on an SQLite database server.

### Usability evaluation of the mobile app

After designing and developing the mobile app, a usability evaluation was carried out. The twenty-five (n = 25) patients diagnosed with Covid 19 that were not required to be hospitalized participated in this part. The demographic characteristics of the users participating in the evaluation stage are shown in Table 1. Most of the infected patients were female with 56% (n = 14) and the most frequent infection with Covid-19 had been for at least a week (n = 13). Table 2 shows users’ opinions on self-care mobile application usability and user satisfaction. The average above 6 was obtained in all assessed dimensions. Thus, the users believed that the application’s usability was at a “good” level.

**Table 1:** Demographic characteristics of the users participating in the Usability Evaluation Stage

Characteristics	Groupe	Number	Percent (%)
Age	> 60	3	12%
	< 60	22	88%
Gender	Female	14	56%
	Male	11	44%
Education level	Diploma and Under Diploma	8	32%
	BSc	12	48%
	MSc and PhD	5	20%
Duration of infection with covid	Less than a week	10	40%
	A week	13	52%
	One to two weeks	2	8%

**Table 2:** Usability Evaluation and and user satisfaction of Covid-19 mobile App

Aspects	Mean (± SD)
Overall reaction to the app	8.15 (± 0.74)
Screen	7.44 (± 1.4)
Terminology and information used in application	8.24 (± 0.76)
Leaning	8.07 (± 0.84)
App capabilities	7.38 (± 1.71)
Total	7.91 (± 1.24)

## Discussion

Mobile-based self-care apps provide an opportunity for patients to actively participate in managing their conditions and changing their behavior and lifestyle to achieve positive health outcomes. With the unprecedented growth of mobile technology, the smartphone plays an important role in supporting chronic disease care. In recent years, when COVID-19 disease spread as an epidemic, people who did not need to be hospitalized had to follow the healthcare guidelines. The aim of the current study was to design develop and evaluate a self-care mobile app for people with COVID-19 who were quarantined at home. The mobile app design consisted of two main parts: the user registration page and the main home page. The main menu includes (1) COVID-19 educational topics, (2) COVID-19 self-care instructions (3) COVID-19 symptom management, and (4) Medications Reminders and Alarms. Te evaluation results indicated that the application was at a good level of usability with an average score of 7.91 ± 1.24.

In recent years, the use of mobile-based applications has increased in various areas of health due to their development and low cost. Software-based self-care behaviors have been reported to be successful in providing effective health care [54–57]. Self-care behaviors at home are the best solution to control COVID-19 due to the unknown behaviors of coronavirus and the lack of approval of antiviral drugs in the treatment of this disease [16, 58, 59]. Despite the potential of m-Health interventions for improving infectious disease management, in a systematic review by Lee et al., a significant inconsistency in the outcome measures reported in Tuberculosis (TB) mHealth literature in low- and middle-income countries (LMICs) was reported [60]. Zamberg et al. indicated that the use of the mobile-based application seemed to be a cost-effective and time-saving way during the COVID-19 pandemic [12].

The present study designed a mobile app based on Android that had functions such as educational topics, self-care instructions, symptom management, medication reminders, and other activities. Previously Mehraeen et al., represented that the use of these capabilities can improve self-care and medication adherence, resulting in the promotion of antiretroviral etherapy in human immunodeficiency virus [56]. In the various studies with the aim of designing and use of applications in infectious diseases, features such as adherence to medications, reminders, and symptom management have been considered by designers of applications [61–63]. In this regard, Saeednia et al. in designing a mobile-based self-care application for patients with COVID-19 have applied functions such as medication reminders, exercise, diet, and educational topics [61]. Timmers et al., in an observational cohort study, established the successful performance and use of an application with COVID-19 education, self-assessment, and symptom tracking [11]. Moreover, We did implement reminders and notifications for regular consumption of prescribed medicines and educational topics, which is in accordance with the findings of a study by Nikan et al. regarding designing a self-care application for patients with acquired immune deficiency syndrome [56]. Safdari et al., in a mobile-based self-care system (based on an android system) for TB management, considered adherence to drugs, drug consumption reminders, and educational messages for patients [64]. Poor adherence to the prescribed medicines contributes to the significant deterioration of disease management, death, and elevated health care costs [65]. Therefore, innovative approaches such as mobile-based application reminders and controllers with a charming user-centered interface can assist patients to increase adherence to the prescribed drugs [66, 67].

Access to the latest information and instructions related to COVID-19 are important practices that can help implement self-care guidelines. In other words, access to essential information and awareness of the latest disease statistics can help create awareness about infection control. In chronic conditions that require community participation to control them, providing information to the population plays a key role in managing critical situations. Also, the timely provision of accurate information in serious conditions will be a pioneer in the implementation of international guidelines. In this developmental research, people can access the latest information and instructions related to COVID-19 and share this information using mobile health applications. Through educational content in the design of self-care applications, patients acquired knowledge and skills needed to self-care for their disease [68–70]. In the study by Saeednia et al., educational messages were one of the parts of designing the COVID-19 self-care application [61]. Mehraeen et al. reported that providing educational information, proper diet, and exercise programs gradually changed diabetic people’s behaviors, improved their lifestyles, and increased patients’ motivation to continue treatment [21]. Swendeman and Perera considered educational information in self-care applications for HIV infection [71, 72]. Narasimhan et al., have also applied educational messages about TB and removed the stigma related to their disease in a self-care application for TB patients [73]. The usability test by users of mobile apps indicates that the app is a good level of satisfaction. Saeinia et al., conducted a review of the usability test of mobile apps during the COVID-19 pandemic and indicated the usability testing method uses representative users to test how easy it is for a design to be used [74]. In this study, we had two limitations, first, the lack of mobile-based health applications for COVID-19 in Iran. Another limitation was the lack of information to design a user-centered interface based on the features and requirements of the mobile application.

## Conclusion

The COVID-19 is still disturbing numerous people everywhere in the world. Symptoms of COVID-19 are mild and treatable in 80% of infected people and do not require hospitalization. In general, people should follow self-care strategies to reduce the risk of transmission. In this study, a mobile-based self-care application was developed and evaluated for patients with COVID-19 who are in quarantine at home. This app can be a guideline for managing symptoms, reminding medications, and implementing self-care instructions in outpatients. Developing and evaluating the mobile apps can be validated by ensuring usability tests throughout the production process. It is recommended that the effectivness test of these apps be investigated by researchers in the next studies. The authors suggest evaluating the efficacy and functionality of mobile-based applications for COVID-19 in clinical trial studies.

## Data Availability

The datasets generated during and analyzed during the current study are not publicly available due to including a series of specialized information related to the type of application design but are available from the corresponding author on reasonable request.

## Abbreviations

TB: Tuberculosis TB
LMICs: Low- and middle-income countries
AOS: Android Operating System
OOB: Object-Oriented Programming
PHR: Personal Health Record
IDE: Integrated Development Environment
XML: Extensible Markup Language
